# The Use of Wearable Inertial Sensors and Workplace-Based Exercises to Reduce Lateral Epicondylitis in the Workstation of a Textile Logistics Center

**DOI:** 10.3390/s23115116

**Published:** 2023-05-27

**Authors:** Florian Michaud, Roberto Pazos, Urbano Lugrís, Javier Cuadrado

**Affiliations:** 1 Laboratory of Mechanical Engineering, Campus Industrial de Ferrol, Universidade da Coruña, 15403 Ferrol, Spain; urbano.lugris@udc.es (U.L.); javier.cuadrado@udc.es (J.C.); 2 The Well-Being Lab S.L., 15005 A Coruña, Spain; roberto.p@thelab.com.es

**Keywords:** movement analysis, kinematics, motion capture, wearable sensors, workplace-based exercise, musculoskeletal disorders, well-being, epicondylitis, ergonomics

## Abstract

People whose jobs involve repetitive motions of the wrist and forearm can suffer from lateral epicondylitis, which is a significant burden on both the individual and the employer due to treatment costs, reduced productivity, and work absenteeism. This paper describes an ergonomic intervention to reduce lateral epicondylitis in the workstation of a textile logistics center. The intervention includes workplace-based exercise programs, evaluation of risk factors, and movement correction. An injury- and subject-specific score was calculated from the motion captured with wearable inertial sensors at the workplace to evaluate the risk factors of 93 workers. Then, a new working movement was adapted to the workplace, which limited the observed risk factors and took into account the subject-specific physical abilities. The movement was taught to the workers during personalized sessions. The risk factors of 27 workers were evaluated again after the intervention to validate the effectiveness of the movement correction. In addition, active warm-up and stretching programs were introduced as part of the workday to promote muscle endurance and improve resistance to repetitive stress. The present strategy offered good results at low cost, without any physical modification of the workplace and without any detriment to productivity.

## 1. Introduction

Repetitive motions of the wrist and forearm can lead to lateral epicondylitis (LE), a painful musculoskeletal disorder (MSD) also known as tennis elbow, caused by inflammation of the tendons that attach the forearm muscles to the elbow [1]. Rest and conservative treatments often help relieve the pain, while, in a small percentage, surgery is necessary [2]. People whose jobs involve this type of movement are at risk of this injury [3,4,5,6,7], which is a significant burden on both the individual and the employer due to treatment costs, reduced productivity, and work absenteeism [8,9,10]. In this study, the Occupational Risk Prevention Department (ORPD) of the logistics center of a textile company reported numerous LE cases (12 workers diagnosed with long-term illness in the three years prior to this intervention) at a manual workplace in which the worker has to take a determined number of garments from a cardboard box and place them, one by one, on an automated distribution line. The ORPD showed concern for the health of the workers and requested an ergonomic intervention to reduce work-related musculoskeletal disorder (WMSD).

The purpose of ergonomic interventions is to help answer various questions related to the safety of employees, working conditions, and performance of the system. In the working environment, their aim is to fit the job to the worker and they are categorized into three specific factors: physical, organizational, and psychosocial factors, which can be combined [11,12]. The first one focuses on the biomechanical risk factors such as repetition, forceful exertion, awkward postures, manual materials handling, and vibration [13,14,15,16]. Organizational factors such as short cycle times, unbalanced task content, insufficient recovery time, collaboration, and support could also contribute to the development of WMSDs in workplaces [17,18]. The psychosocial aspects of work such as high job demands, low job control, poor social support, and inadequate communication at work have been recognized to increase the risk of MSDs. These factors can lead to increased stress, tension, and fatigue, which in turn can increase the likelihood of developing MSDs [19]. The interaction between the task and the people is usually evaluated through multidimensional methods such as observational-based tools, questionnaires, or direct measurements [20]. Nowadays, technological advances and, in particular, wearable devices offer an objective assessment of physical factors [21,22,23,24,25,26,27,28,29].

In the workplace considered in this paper, previous interventions had been carried out before. An organizational ergonomic intervention limited the time spent in the mentioned workplace to two hours per day. Then, workers had to switch to another workplace (after a rest time) which entailed a different movement and effort. At the cognitive level, the task was simplified: a screen indicates in real time to the worker how many garments must be placed on the horizontal carousel, so that the task is easy and the work environment is free of disruptions. However, despite these modifications, LE cases did not decrease, and a physical ergonomics intervention was not possible for economic and logistical reasons.

For these reasons, the intervention proposed in this study to reduce MSDs at the workplace was composed of:

- An individual evaluation, using wearable sensors, of the working movement of the 93 workers who occupied the workplace.
- An individual interview to inform each worker on the risk observed according to their evaluation and to discuss their personal physical limitations through a questionnaire.
- Personalized training sessions with a personal trainer to adopt a less harmful work movement adapted to the worker.
- A second individual evaluation of the corrected working movement using wearable sensors to validate the approach with a sample of 27 workers.
- A workplace-based exercise intervention composed of daily warm-up and stretching exercises.

Workplace-based exercises resulted in improvements in work ability and allowed the workers to meet the demands of the work considering their health, personal resources, and work environment [30,31]. They were compatible with the constraints imposed by the company (physical and economic) and complemented the intervention well. The strategy offered good results at low cost, without a physical modification of the workplace and without any detriment to productivity.

## 2. Material and Methods

### 2.1. Participants, Workplace, and Task Description

93 workers who occupied the workplace (with no exclusion criteria) were recruited for this study; their characteristics are reported below:

- A total of 61 males and 32 females;
- Age: 40 ± 18 years;
- A total of 85 right-handers and 6 left-handers.

**Figure 1:**
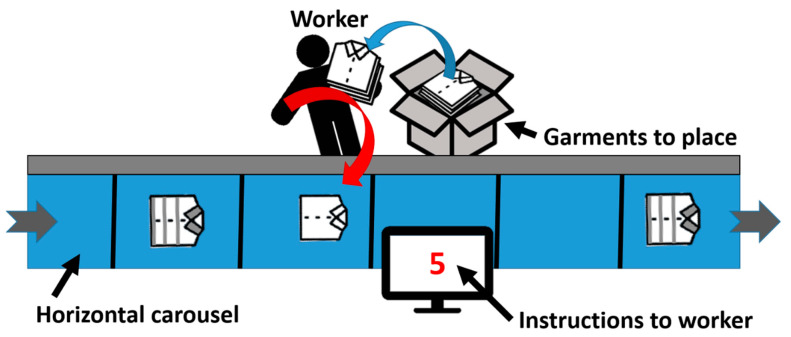
Workplace and task illustration.

The work shift of the logistics center was defined in such a way that workers were assigned to this workplace for two hours throughout their workday and to other workplaces during the rest of the time. As illustrated in Figure 1, the task consisted of taking the garments from the boxes, which were continuously and automatically supplied to each worker of the chain, and placing the garments, one by one, into an empty basket (40 cm × 50 cm) of the horizontal carousel passing at a constant velocity of 1 m/s. A screen situated in front of the worker indicated, in real time, the number of garments that the worker had to put on the carousel from the corresponding box. Until this intervention, workers were free to choose how to execute the task, although most employees were observed to follow the same procedure: pick up some items, carry them with their nondominant arm and the elbow flexed, and throw them onto the carousel with their dominant hand. Finally, it must be added that the type, number, size, and weight of the garments were random, had great variability, and influenced the execution of the task.

During the three years prior to the intervention, a total of 12 workers were diagnosed with LE and had to be absent for long periods of time due to long-term illness.

### 2.2. Intervention

**Figure 2:**
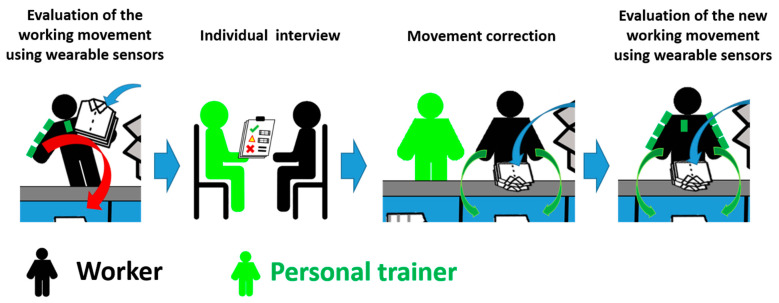
Working movement correction intervention.

As illustrated in Figure 2, the intervention proposed in this study to reduce MSDs at the workplace was composed of:

- An individual evaluation, using wearable sensors, of the working movement of the 93 workers who occupied the workplace.
- An individual interview to inform each worker on the risk observed according to their evaluation, and to discuss their personal physical limitations through a questionnaire.
- Personalized training sessions with a personal trainer to adopt a less harmful work movement adapted to the worker.
- A second individual evaluation of the corrected working movement using wearable sensors to validate the approach with a sample of 27 workers.

The mentioned steps were complemented with a workplace-based exercise intervention, composed of daily warm-up and stretching exercises, which are described in detail below.

#### 2.2.1. Experimental Data Collection

**Figure 3:**
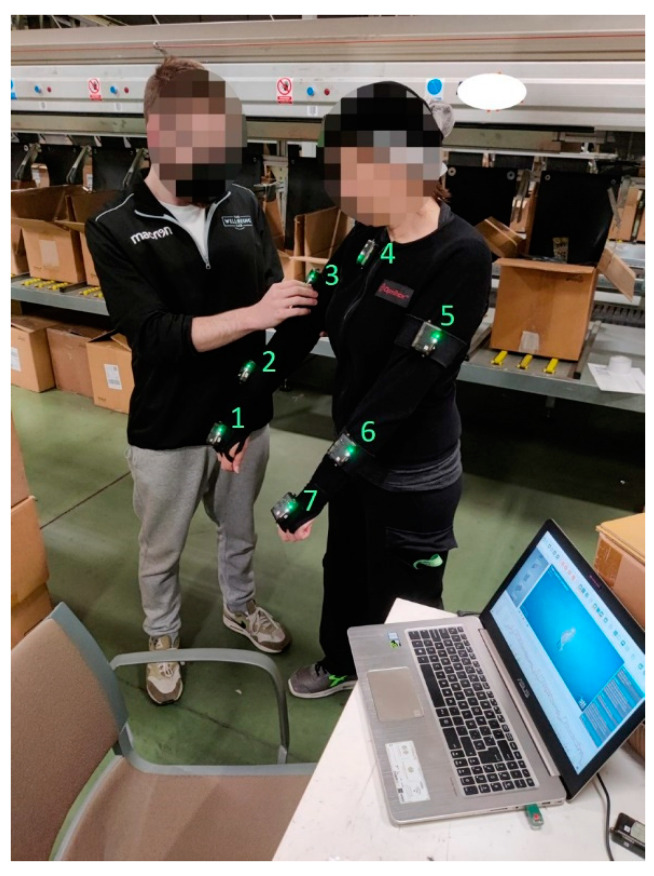
Wearable system for motion capture at the workplace (sensors numbered in green).

Individual measurements of the 93 workers were conducted in multiple sessions on different work shifts and days at the same workplace. In order to disturb the company’s activity as little as possible and work under real working conditions, the measurements were made in the logistics chain during a normal working day. The workers were individually equipped with a motion-tracking jacket (typically used for optical motion capture and available in four different sizes) and 7 inertial measurement units (IMUs) (NGIMU, X-io Technologies Limited, Bristol, United Kingdom [32]) sampling at 100 Hz, placed at trunk, arms, lower arms, and hands, to measure upper limb movements (Figure 3). Orientations from IMUs and joint kinematics were computed by commercial software *iSen* 2019.0 (STT Systems, San Sebastian, Spain) [33]. A trial session was carried out at the workplace to ensure that measurements from IMUs would not be affected by ferromagnetic materials present in the environment [23,34]. Velcro straps were added over the sensors positioned on the jacket to reduce motion artifacts. Before commencing the motion capture, the subjects performed a static pose for the calibration of the sensors and carried out a series of movements to ensure that the sensors attached to their bodies did not hinder performance and did not show motion artifacts. Then, the workers were asked to carry out their daily tasks as usual during two series of loading and unloading of garments (between 10 and 30 repetitions depending on the worker and the sizes of the items) with two different types of garments. Because the type, number, size, and weight of the garments were random and had great variability, it was impossible to use the same item to guarantee objective comparisons between subjects. However, products which require a totally different movement and which are rarely encountered (e.g., coats) were not considered in this study. All subjects gave written informed consent for their participation.

#### 2.2.2. Work-Related Evaluation of Risk Factors

While the use of wearable devices for motion capture offers objective measurements of the worker, occupational physicians need to interpret these data to evaluate the worker’s exposure risk factor. Risk factors are related to the workstation, the task, and the worker behavior, but overall to the pathology. Because the aim of this ergonomics intervention was to reduce LE cases at the particular workplace, it was necessary to first have a good understanding of this MSD.

LE is the result of the overuse of the wrist extensor muscles, leading to inflammation or irritation of the tendon insertion [1,5,35]. The sum of posture, force, and repetitiveness configures the fatigue cycle ending up in repetitive trauma, which causes insufficient vascular circulation in the soft tissues, inflammation in the tendons due to excessive friction in the anatomical corridors, compression of the nerves as a consequence of the inflammation of the muscles, ligaments, and tendons, and instability of the joints due to forced postures, injuries, or breaks of the soft tissues [36]. Repeated microtraumas cause severe pain at the attachment sites of the extensor tendons on the lateral side of the elbow joint [35]. Manual labor with dominant-side involvement, grasping of objects, repetitive bending and straightening of the elbow, repetitive bending of the wrist, high percentage of time spent in hand supination or pronation of more than 45°, and high wrist angular velocity were reported as specific exposures to LE [3,4,5,6,7].

Ergonomic risk scores allow a simplified data interpretation, but they are often associated with a workstation, with postures instead of motions, and ignore the variability among operators [28]. In this study, we decided to create a specific ergonomic risk evaluation associated with LE to evaluate all the workers individually, but above all, to allow them a better understanding of the measures in order to offer them personal recommendations. Based on LE’s risk factors previously reported, a risk score was specifically designed, for which 40 points were distributed as follows:

(1) 15 points for the hand and forearm that throw the garment (arm 1).
  (a) % of time spent with hand supination or pronation of more than 45°: 5 points.
  (b) Wrist extension amplitude: 5 points.
  (c) Wrist extension angular velocity: 5 points.
(2) 10 points for the elbow of the arm 1.
  (a) Elbow 1 extension amplitude: 5 points.
  (b) Elbow 1 extension angular velocity: 5 points.
(3) 10 points for the rest of the joints. % of time spent out of the recommended comfort angles [36].
  (a) Shoulder 1: 3 points (1 point for each angle).
  (b) Shoulder 2: 3 points (1 point for each angle).
  (c) Elbow 2: 1 point.
  (d) Wrist 2: 3 points (1 point for each angle).
(4) 5 points for the task frequency.

The time periods that corresponded to garment throws were manually identified and used for the evaluation because the intervention focused on the specific activity. Special attention was paid at the wrist and elbow level of the most active hand, although the rest of the joints were also considered in the study. For the “% of time” evaluations in the list above, the maximum score was assigned to 50%. For (1b), the maximum score was assigned to a mean angle of 45°. For (2a), the maximum score was assigned to a mean angle of 30°. For (1c) and (2b), the maximum score was assigned to maximum throwing velocities of 200°/s. In all cases, the scores were linearly scaled for lower values of the inputs. Moreover, to gather the difference between the loaded and unloaded condition of the second arm, the scores for the second arm were divided by 2 if the worker did not load it. Finally, because the company records the workers’ activity, it was preferred to use this information to more objectively determine the task frequency along the work shift: the score was linearly scaled between 10 and 25 repetitions per minute [37].

#### 2.2.3. Movement Correction

After the evaluation of individual risk factors, an interview was organized with each worker to inform the participant on the risk observed according to their way of executing the task and to discuss their personal physical limitations through a questionnaire (previous pathologies, specific pain during the task, etc.), in order to adapt the movement to the participant. Although in activity ergonomics, researchers are not in favor of postural corrections and prefer to carry out physical ergonomics interventions, it must be recognized that a certain task may present different levels of risk depending on the person who executes it and the posture adopted (such as the manual handling of loads) [23]. 

**Figure 4:**
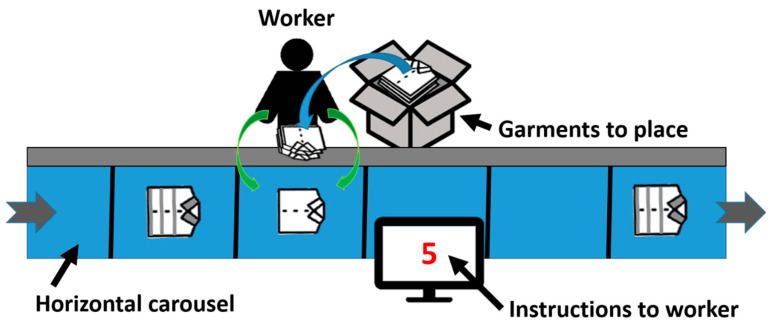
Corrected task illustration.

The risk factors can be reduced by decreasing the joint angles, range of motion, frequency, and velocities. Therefore, some tips were taught by a specialist in sport and physical activity to the workers during 30-minute personalized training sessions (two per week over five weeks) at their own workplace. The general recommendations were: (a) take each pile of garments from the box with both hands; (b) use the structure of the carousel (Figure 3 in grey) to put the pile of garments on it (with the body facing the carousel and the back in the neutral position); (c) use one hand to keep the pile of garments on the structure and use the second hand to put each garment on the carousel; (d) avoid taking and throwing the product with hand pronation or supination; (e) use the full arm to throw the item (shoulder, elbow, and wrist) and not only the wrist; (f) alternate the hands’ task every 10 minutes (Figure 4). The latter was the most difficult for the workers to learn because the dominant hand is stronger, faster, and, above all, more dexterous, but it is the only way to reduce the frequency of movements without detriment to productivity. Because the task was relatively easy, after a few sessions, the workers were able to execute the task at almost the same speed that with their dominant hand.

One month after the training, a sample group of 27 workers (among the 93 workers) was selected to check whether they had reduced their risk factors. For an objective comparison, the same garments were used. In order to reflect that workers were using the structure of the workstation to support the piles of garments instead of holding them in their second arm, the scores of the second arm were divided by 2.

#### 2.2.4. Data Analysis

Histories of upper-extremity joint angles were computed using the commercial software iSen (STT Systems, San Sebastian, Spain) [33], based on the IMU measurements. Measurements associated with the previously reported evaluation of risk factors (see Section 2.2.2) were processed and assessed for all the subjects, using a custom-made Matlab program.

In this study, statistical analysis was employed to analyze gender differences and intervention improvements. To compare gender differences, a two-sample t-test was applied since it involved comparing two different groups on the same measure. However, to analyze intervention improvements, the paired t-test was chosen because it involved rating the same participants on the same measure at two different time points (before and after the intervention) [38]. The statistical analysis was performed using a custom-made Matlab program that implemented the mentioned methods [38].

#### 2.2.5. Workplace-Based Exercises

**Figure 5:**
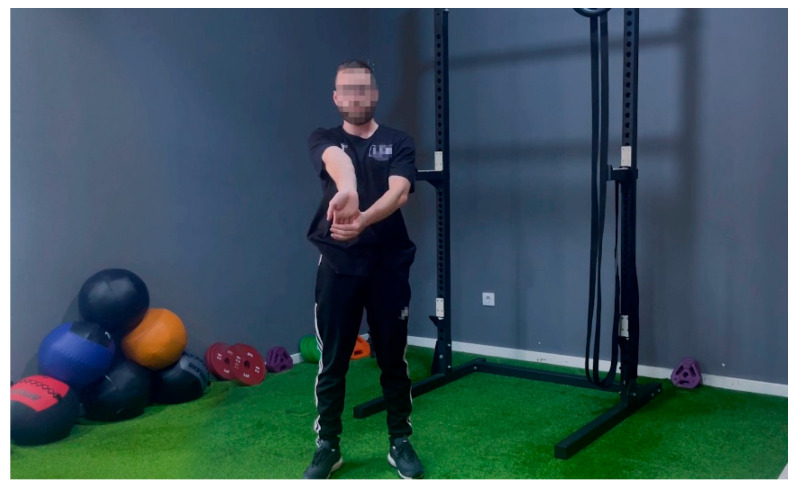
Recorded training instructions.

The early goal of a therapeutic exercise program is to promote muscle endurance and improve resistance to repetitive stress injuries. In addition, specific exercises to stretch and strengthen the muscles attached to the injured tendon will help with the healing process [39,40]. For this reason, a room for carrying out basic gym exercises was set into the logistics center so that the training sessions could be part of the working day. First of all, the employees received a talk to raise awareness about the usefulness and necessity of these exercises for their well-being. Then, over eight weeks, a gym trainer gave short (5 min) group training sessions at the beginning and end of each work shift. The training sessions were recorded and projected on the TV present in the room (TV image shown in Figure 5) to help the workers follow the program.

The session before the task was devoted to an active full-body warm-up, which serves to increase soft tissue temperature and enhance muscle performance prior to an activity [41]. The exercises are simple, easy to understand for all people, and do not require any specific tools:

- Four neck internal and external rotations.
- Four neck tilts (for each direction).
- Ten chest expansions.
- Ten wrist circles with clasped hands (for each direction).
- Four standing toe touching with feet shoulder width apart (left foot, center, and right foot).
- Five torso rotations (for each direction).
- Five standing single leg hip rotations (each leg).
- Five bodyweight deep squats hold.
- Five bodyweight standing calf raises.
- Five bodyweight standing toe raises.

The session after the task is composed of static stretching exercises, that are recommended for improving recovery of strength and range of motion, and also, for reducing delayed onset muscular soreness after physical exertion [42]. Again, the exercises are simple, easy to understand, for all people, and do not require any specific tools:

- three standing toe touch stretches with feet shoulder width apart (holding 3 s for each foot).
- One standing quadriceps stretch (holding 8 secs for each leg).
- One standing knee to chest stretch (holding 8 secs for each leg).
- Two standing arms backward chest stretch (holding 2 s).
- Two standing side bend stretches (for each direction).
- Three standing wrist prayer stretches.
- Three open-hand finger stretches (holding 2 s).
- Three wrist extensor stretches (holding 2 s for each hand, Figure 5).
- Two wrist flexor stretches (holding 2 s for each hand).
- Three neck flexion stretches (holding 2 s).

## 3. Results

### 3.1. Pre-Intervention

The mean scores of the 93 operators (two recordings per worker were taken before the intervention) are reported in Table 1, showing a mean total score of 22.81 out of 40. Based on these values, it can be said that major risks come from wrist extension, elbow extension, and highly repetitive tasks. The standard deviation was calculated and reflects the high inter-subject variability for these parameters. Mean values of the different evaluated risk factors are reported in Table 2. Again, the standard deviation shows a high variability corresponding to the subject-specific motion. As for the task frequency, it was set at 25 repetitions per minute for all the subjects based on company records (mean of 1500–1700 garments per hour and per worker).

**Figure 6:**
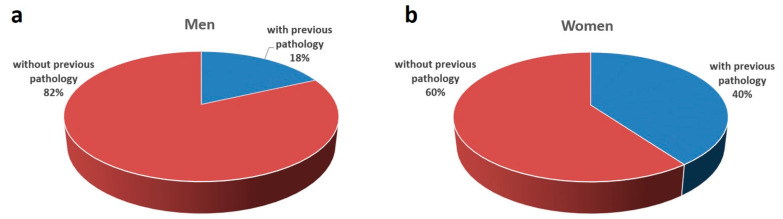
Workers with previous pathologies in wrist or elbow according to gender: (**a**) men; (**b**) women.

Because several studies highlighted that LE is more common in women [3,4,43], special attention was paid to gender differences. The results of the questionnaire revealed that 18% of men suffered from pathologies in wrist or elbow before the intervention, compared to 40% of women (Figure 6).

Table 3 shows that the mean scores of the 61 men and the 32 women were 21.70 and 24.93 out of 40, respectively, indicating higher risk factors associated with wrist and elbow extension for women. A two-sample t-test was applied to compare the differences between the two genders, which demonstrated that women had significantly higher scores for elbow and global risk factors than men, with a confidence level higher than 99.9%.

### 3.2. Post-Intervention

The comparison of the average scores achieved by the 27 workers measured before and after the intervention is reported in Table 4. The mean total score moved from 23.87 to 14.96, which corresponds to a reduction of more than 37%. All the risk factors were significantly improved, and paired sample t-tests demonstrated that the total and main risk factors were statistically reduced (*p* < 0.06). The mean values of the corresponding scores are reported in Table 5. All the values for the risk factors were improved except the percentage of time spent out of the recommended comfort angles for the elbow of the second arm. However, the score for the latter was reduced as workers no longer carried the pile of garments with this arm. The calculated p-values demonstrated that the main risk factors related to wrist and elbow extension were statistically improved, with p-values greater than 0.15 and 0.001, respectively. By using both hands to perform the task, task frequency was cut in half without affecting the company’s production, and the corresponding score was significantly reduced.

The questionnaire given to the 93 workers at the end of the training revealed 100% satisfaction. At the physical level, they felt a great difference and they were less tired thanks to the intervention, particularly because they no longer had to carry the load of the pile of garments with their second arm. Last, but not least, the ORPD has not reported new cases of LE in the past year, the intervention having started a year and a half ago.

## 4. Discussion

This paper presents an ergonomic intervention to reduce LE at a workstation in the textile industry, composed of workplace-based exercise programs, evaluation of risk factors, and correction of the working movement. The application of wearable devices and motion analysis highlighted the possible causes of the injuries at the workplace considered. Significant angle amplitudes and high angular velocities for wrist and elbow extensions were observed, which, combined with high task frequencies, can result in LE [3,4,5,6,7]. The creation of a subject-specific injury score for evaluating risk factors, combined with a personal interview and personalized training, improves the consideration of the employees as individuals, which is certainly the main reason of their satisfaction and the reduction in injuries. Although the task seemed simple, significant variations were found in the way the task was executed by the workers. The evaluation revealed that significant differences were observed between men and women, with women presenting higher scores. These results could explain why more women in the workplace reported previous pathology in wrist and elbow in the questionnaire and why other studies highlighted that LE is more common in women [3,4,43]. Despite the differences between workers, all the risk factors were statistically reduced by teaching a new way to execute the task. Risk factors remained significant at the wrist and elbow levels but were difficult to reduce under the workplace and task constraints. The alternative use of hands to complete the task was found to be a good strategy to significantly reduce repetitive strain injuries without detriment to productivity [44]. The reduction in injuries demonstrated the benefit of this intervention, which included movement correction and a workplace-based exercise program. The partial effects of the different parts of the ergonomic intervention cannot be dissociated because all the workers received the full intervention. The effectiveness of resistance and stretching training programs in the workplace for the prevention of upper extremity MSD was already reported in previous studies [45,46,47]. As a general limitation of this study, because the initial objective was to reduce MSD at work (and not to conduct research), this intervention was applied to all the workers in this workplace and no control group was considered, with the effectiveness of this intervention having been demonstrated in previous studies [30,31]. Finally, we are aware that, in activity ergonomics, researchers are not in favor of posture corrections and prefer to address physical ergonomics interventions. However, for economic and logistical reasons, the logistics center of the textile company was strict and did not allow physical modifications of the workplace. Despite this limitation, the posture correction coupled with workplace-based exercises offered good results at a low cost without physical modification of the workplace and without detriment to productivity.

As a future work, the authors would like to apply their recent advances in the real-time estimation of muscle efforts [48,49] and fatigue [50] using computer simulations, to evaluate working movements so as to further improve injury prevention and risk evaluation. Additionally, it is hypothesized that optimal-control-based predictive simulation, which is used to predict and optimize treatment outcomes [51,52], could be applied to find harmless working movements within workplace limitations.

## 5. Conclusions

Analysis and correction of the working movement using wearable inertial sensors coupled with workplace-based exercise intervention were found to be a good strategy to reduce LE in the target workplace. The proposed approach could serve as a complement to physical ergonomics interventions or as an alternative when they are not possible.

## Figures and Tables

**Table 1:** Mean scores and standard deviations of the 93 workers prior to the intervention.

Risk Factors			Mean Scores	SD	Maximum
Active arm	Wrist	Supination/pronation >45° (%time)	0.17	0.48	5
		Extension amplitude (°)	2.83	1.04	5
		Extension velocity angle (°/s)	4.19	1.05	5
	Elbow	Extension amplitude (°)	3.46	1.36	5
		Extension velocity angle (°/s)	4.00	1.30	5
	Shoulder	Comfort angle flexion/extension (%time)	0.46	0.40	1
		Comfort angle lateral elevation (%time)	0.41	0.41	1
		Comfort angle internal rotation (%time)	0.05	0.13	1
Second arm	Wrist	Comfort angle flexion/extension (%time)	0.07	0.19	1
		Comfort angle deviation (%time)	0.62	0.39	1
		Comfort angle pronation/supination (%time)	0.14	0.27	1
	Elbow	Comfort angle flexion/extension (%time)	0.00	0.02	1
	Shoulder	Comfort angle flexion/extension (%time)	0.07	0.22	1
		Comfort angle lateral elevation (%time)	0.82	0.60	1
		Comfort angle internal rotation (%time)	0.52	0.87	1
Task frequency (rep/min)			5.00	/	5
Total			22.81	4.35	40

**Table 2:** Mean values and standard deviations of the 93 workers prior to the intervention.

Risk Factors			Mean Values	SD
Active arm	Wrist	Supination/pronation >45° (%time)	1.73	4.83
		Extension amplitude (°)	25.57	9.53
		Extension velocity angle (°/s)	187.49	64.43
	Elbow	Extension amplitude (°)	71.55	15.62
		Extension velocity angle (°/s)	186.00	81.21
	Shoulder	Comfort angle flexion/extension (%time)	29.36	30.68
		Comfort angle lateral elevation (%time)	27.53	32.52
		Comfort angle internal rotation (%time)	2.67	7.66
Second arm	Wrist	Comfort angle flexion/extension (%time)	3.58	10.40
		Comfort angle deviation (%time)	41.15	32.31
		Comfort angle pronation/supination (%time)	8.16	18.41
	Elbow	Comfort angle flexion/extension (%time)	0.12	1.23
	Shoulder	Comfort angle flexion/extension (%time)	4.98	17.85
		Comfort angle lateral elevation (%time)	41.04	29.90
		Comfort angle internal rotation (%time)	13.05	21.67
Task frequency (rep/min)			25.00	/

**Table 3:** Mean scores and standard deviations of men and women prior to the intervention (*p* < 0.05 in red).

Risk Factors			Men		Women		*p*-Value
			Mean Scores	SD	Mean Scores	SD	
Active arm	Wrist	Supination/pronation >45° (%time)	0.19	0.52	0.14	0.40	0.31
		Extension amplitude (°)	2.75	1.04	2.99	1.03	0.14
		Extension velocity angle (°/s)	4.12	1.12	4.33	0.91	0.18
	Elbow	Extension amplitude (°)	3.04	1.38	4.25	0.86	0.00
		Extension velocity angle (°/s)	3.60	1.40	4.75	0.57	0.00
	Shoulder	Comfort angle flexion/extension (%time)	0.37	0.38	0.64	0.38	0.00
		Comfort angle lateral elevation (%time)	0.35	0.40	0.52	0.43	0.04
		Comfort angle internal rotation (%time)	0.04	0.12	0.08	0.14	0.09
Second arm	Wrist	Comfort angle flexion/extension (%time)	0.06	0.19	0.08	0.21	0.37
		Comfort angle deviation (%time)	0.53	0.40	0.78	0.30	0.00
		Comfort angle pronation/supination (%time)	0.09	0.22	0.22	0.34	0.03
	Elbow	Comfort angle flexion/extension (%time)	0.00	0.03	0.00	0.00	0.20
	Shoulder	Comfort angle flexion/extension (%time)	0.09	0.26	0.04	0.11	0.09
		Comfort angle lateral elevation (%time)	0.93	0.62	0.61	0.50	0.01
		Comfort angle internal rotation (%time)	0.53	0.90	0.50	0.80	0.44
Task frequency (rep/min)			5.00	0.00	5.00	0.00	/
Total			21.70	4.56	24.93	2.95	0.00

**Table 4:** Mean scores and standard deviations of the 27 workers prior and posterior to the intervention (*p* < 0.05 in red).

Risk Factors			Pre		Post		*p*-Value
			Mean Scores	SD	Mean Scores	SD	
Active arm	Wrist	Supination/pronation >45° (%time)	0.31	0.58	0.25	0.49	0.18
		Extension amplitude (°)	2.98	0.81	2.56	1.12	0.06
		Extension velocity angle (°/s)	4.39	0.77	3.96	1.02	0.03
	Elbow	Extension amplitude (°)	3.62	1.13	2.63	1.18	0.00
		Extension velocity angle (°/s)	4.32	0.98	3.27	1.09	0.00
	Shoulder	Comfort angle flexion/extension (%time)	0.45	0.39	0.30	0.30	0.01
		Comfort angle lateral elevation (%time)	0.40	0.45	0.13	0.22	0.00
		Comfort angle internal rotation (%time)	0.01	0.03	0.00	0.00	0.09
Second arm	Wrist	Comfort angle flexion/extension (%time)	0.06	0.16	0.00	0.01	0.04
		Comfort angle deviation (%time)	0.61	0.39	0.25	0.33	0.00
		Comfort angle pronation/supination (%time)	0.18	0.28	0.01	0.06	0.00
	Elbow	Comfort angle flexion/extension (%time)	0.02	0.06	0.01	0.09	0.46
	Shoulder	Comfort angle flexion/extension (%time)	0.00	0.02	0.00	0.01	0.24
		Comfort angle lateral elevation (%time)	0.92	0.60	0.43	0.55	0.00
		Comfort angle internal rotation (%time)	0.60	0.73	0.16	0.65	0.00
Task frequency (rep/min)			5.00	0.00	1.00	0.00	0.00
Total			23.87	3.58	14.96	3.82	0.00

**Table 5:** Mean values and standard deviations of the 27 workers prior and posterior to the intervention (*p* < 0.05 in red).

Risk Factors			Pre		Post		*p*-Value
			Mean Values	SD	Mean Values	SD	
Active arm	Wrist	Supination/pronation >45° (%time)	3.09	5.82	2.45	4.89	0.18
		Extension amplitude (°)	26.83	7.31	23.40	10.90	0.09
		Extension velocity angle (°/s)	196.12	56.30	176.82	72.78	0.15
	Elbow	Extension amplitude (°)	72.44	14.88	51.80	13.56	0.00
		Extension velocity angle (°/s)	186.80	56.04	135.77	54.45	0.00
	Shoulder	Comfort angle flexion/extension (%time)	28.52	30.67	16.05	17.68	0.01
		Comfort angle lateral elevation (%time)	25.69	32.06	6.27	11.11	0.00
		Comfort angle internal rotation (%time)	0.42	1.57	0.01	0.05	0.09
Second arm	Wrist	Comfort angle flexion/extension (%time)	2.76	8.14	0.06	0.32	0.04
		Comfort angle deviation (%time)	40.43	32.35	25.24	22.94	0.00
		Comfort angle pronation/supination (%time)	9.12	14.10	1.16	2.82	0.00
	Elbow	Comfort angle flexion/extension (%time)	0.76	3.21	1.37	4.42	0.29
	Shoulder	Comfort angle flexion/extension (%time)	0.23	1.19	0.11	0.50	0.33
		Comfort angle lateral elevation (%time)	46.00	30.08	42.87	27.53	0.32
		Comfort angle internal rotation (%time)	14.99	18.28	8.15	16.26	0.07
Task frequency (rep/min)			25.00	0.00	13.00	0.00	0.00

## Data Availability

The data sets generated and analyzed during the current study are not publicly available due to company privacy requirements but are available from the corresponding author upon reasonable request.
